# Case Report: First attempt by off-label use of tenecteplase to treat acute extensive portal venous system thrombosis

**DOI:** 10.3389/fcvm.2024.1342529

**Published:** 2024-02-22

**Authors:** Qianqian Li, Ying Piao, Yongguo Zhang, Xingshun Qi

**Affiliations:** ^1^ Department of Gastroenterology, General Hospital of Northern Theater Command, Shenyang, China; ^2^ Department of Medical Oncology, General Hospital of Northern Theater Command, Shenyang, China

**Keywords:** portal venous system thrombosis, thrombolysis, tenecteplase, bleeding, case report

## Abstract

Acute extensive portal venous system thrombosis (PVST) can cause lethal complications. Herein, we have for the first time reported the use of anticoagulation combined with systemic thrombolysis by tenecteplase in a male patient with a diagnosis of acute extensive PVST but without liver cirrhosis. After thrombolytic therapy, abdominal pain obviously alleviated. However, urinary bleeding developed, which was reversible by stopping thrombolytic drugs. Finally, this case developed cavernous transformation of the portal vein without portal venous recanalization. In future, the efficacy and safety of tenecteplase should be explored in acute extensive PVST cases.

## Introduction

Portal venous system thrombosis (PVST) is a common complication of liver cirrhosis and associated with the severity and progression of liver cirrhosis, but is relatively rare in patients without liver cirrhosis (1). PVST is not only located in the main portal vein, but also extends to mesenteric and splenic veins (2). Occlusive PVST can cause intestinal ischemia and portal hypertension related complications, such as gastroesophageal variceal bleeding (3). Anticoagulation is the first-line therapeutic choice in patients with acute PVST, and thrombolysis is considered when this condition is aggravated (4). Thrombolytic agents include streptokinase, urokinase, and tissue type plasminogen activator (t-PA) (5). They are often administered intravenously or locally through a catheter. Tenecteplase is a variant of native t-PA produced by recombinant DNA technology (6). It is approved by the United States Food and Drug Administration for the treatment of acute myocardial infarction (AMI) and is being attempted for the treatment of stroke. To the best of our knowledge, tenecteplase has not been given in cases with PVST yet. Herein, we reported an uncommon case of acute extensive non-cirrhotic PVST who received anticoagulation in combination with systemic thrombolysis by tenecteplase.

## Case presentation

**Figure 1:**
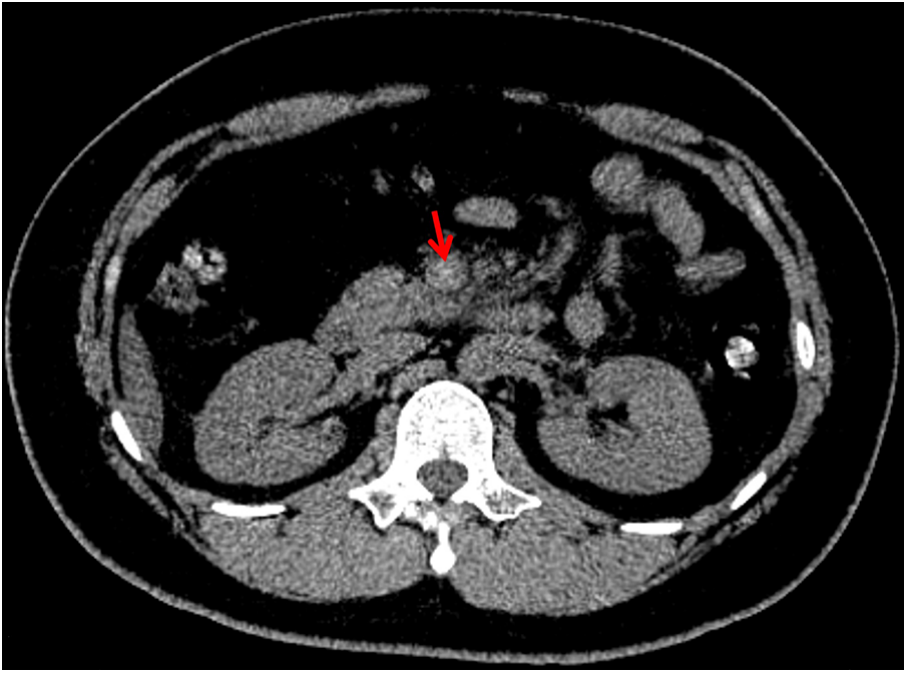
Unenhanced CT image showing high density lesion in superior mesenteric vein.

On July 16, 2023, a 23-year-old male was admitted to our department due to abdominal pain with fatigue and no defecation for a duration of 12 days. He had a history of COVID-19 in February 2023. He had been studying in Australia for 3 years and returned to China on July 15. His body temperature was normal. Physical examination showed upper abdominal tenderness without rebound tenderness. Laboratory tests showed that fibrinogen (FIB) was 7.95 g/L (reference range: 2.00–4.00 g/L), prothrombin time (PT) was 14.7 s (reference range: 9.0–13.0 s), international normalized ratio (INR) was 1.3 (reference range: 8–1.2), C-reactive protein (CRP) was 63.98 mg/L (reference range: <10 mg/L), and total cholesterol was 6.41 mmol/L (reference range: 0–5.17 mmol/L). Intestinal obstruction was excluded by abdominal x-ray scans, but unenhanced computed tomography (CT) scans showed high density lesion in the superior mesenteric vein (Figure 1).

**Figure 2:**
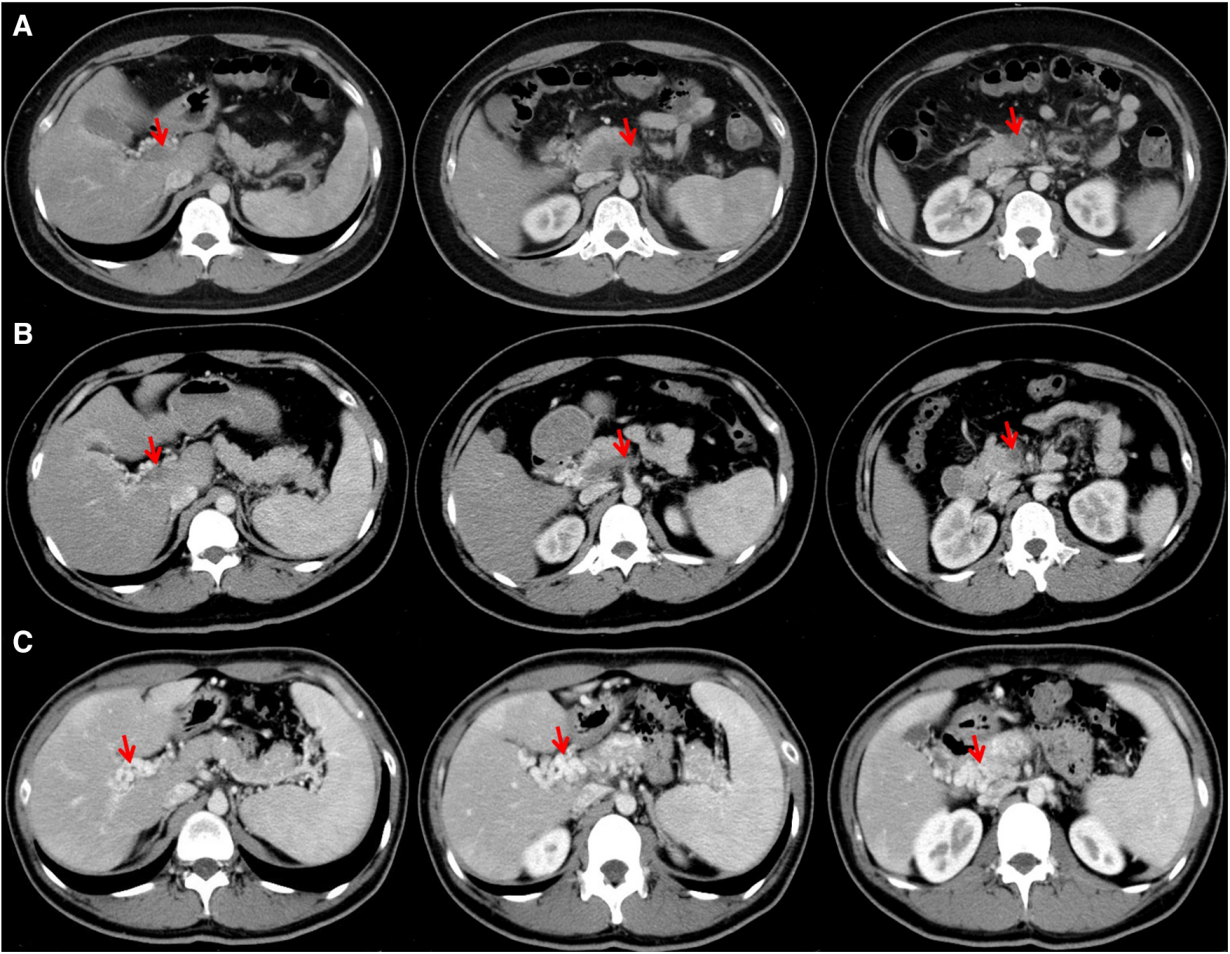
Contrast-enhanced CT images performed on July 17 (**A**) and July 26 (**B**) showing thrombosis in portal vein, splenic vein, and superior mesenteric vein, and those on January 5, 2024 (**C**) showing cavernous transformation of the portal vein.

On July 17, 2023, contrast-enhanced CT scans showed that the portal vein, superior mesenteric vein, and splenic vein thrombus were occlusive (Figure 2A). Gastrointestinal endoscopy showed erosive gastritis without gastroesophageal varices. Laboratory tests showed that D-dimer was 13.47 mg/L (reference range: 0.00–0.55 mg/L), fibrinogen degradation products (FDP) was 30.11 µg/ml (reference range: 0.0–5.0 µg/ml), FIB was 6.36 g/L, PT was 15.3 s, INR was 1.36, CRP was 52.34 mg/L, and procalcitonin (PCT) was 0.14 ng/ml (reference range: 0–0.05 ng/ml). Antithrombin Ⅲ and homocysteine levels were within their reference ranges. Considering a diagnosis of acute extensive PVST, he received subcutaneous injection of enoxaparin 40 mg every 12 h for anticoagulation.

On July 18, 2023, he had a fever with the highest body temperature of 39.5°C, and abdominal pain remained. Laboratory tests showed that CRP was 46.89 mg/L and PCT was 0.27 ng/ml. He received intravenous antibiotics.

On July 19, 2023, his highest body temperature was about 38.5°C. Laboratory tests showed that D-dimer was 15.35 mg/L, FDP was 36.32 µg/ml, FIB was 5.54 g/L, PT was 14.1 s, INR was 1.24, and CRP was 44.56 mg/L. Plasma protein C and S activities were within their reference ranges. He underwent abdominal vascular ultrasound showing extensive thrombosis in the portal vein, superior mesenteric vein, and splenic vein. Thus, continuous intravenous infusion of urokinase 300,000 u every 12 h for systemic thrombolysis was added.

On July 20, 2023, COVID-19 antigen was positive. Laboratory tests showed that antiphospholipid syndrome antibodies were within their reference ranges. His body temperature dropped to 37.9°C, and antibiotic therapy was stopped. To increase the possibility of recanalizing PVST, continuous intravenous infusion of tenecteplase 16 mg every day was employed.

On July 21, 2023, plasminogen activator inhibitor type 1 (PAI-1) gene test showed 4G/5G heterozygous mutation.

On July 22, 2023, a painful patchy ecchymosis with a diameter of 5 cm occurred at the site of enoxaparin injection. Laboratory tests showed significant increases in D-dimer and FDP levels. D-dimer was 40 mg/L, FDP was 104.96 µg/ml, FIB was 5.54 g/L, PT was 13.3 s, and INR was 1.17. Subcutaneous injection of enoxaparin was replaced by 10 mg of rivaroxaban orally twice daily for anticoagulation.

On July 23, 2023, he had gross hematuria about 100 ml in the morning, accompanied by right lumbar discomfort, dizziness, and left upper limb numbness. He was conscious and answered fluently. Physical examination showed the absence of neck stiffness, abnormalities in muscle strength and tension of the limbs, or percussion pain in the renal and ureteral walking areas. Head CT scans did not show abnormal lesions. Routine urine tests showed a large number of red blood cells under the microscope. Anticoagulation and thrombolysis were immediately stopped, and the patient was closely observed.

On July 24, 2023, abdominal pain significantly alleviated without gross hematuria. Laboratory tests showed that D-dimer was 36.38 mg/L, FDP was 58.24 µg/ml, FIB was 4.96 g/L, PT was 13.2 s, and INR was 1.16. Abdominal vascular ultrasound was performed again, which still showed the presence of PVST. After discussing with the patient and his family, anticoagulation and thrombolysis were resumed.

On July 26, 2023, repeated contrast-enhanced CT scans showed that portal vein, superior mesenteric vein, and splenic vein were still occlusive with the development of collateral vessels (Figure 2B). Laboratory tests showed that D-dimer was 5.89 mg/L, FDP was 9.92 µg/ml, FIB was 4.96 g/L, PT was 14.7 s, and INR was 1.3. He discharged with oral rivaroxaban.

At his last visit on January 5, 2024, he did not have any other complaint. Repeated contrast-enhanced CT scans showed that portal vein could not be identified, but was replaced by multiple collateral vessels (Figure 2C).

## Discussion

Gastroenteritis, irritable bowel syndrome, pancreatitis, diverticulitis, appendicitis, and abdominal tumors are common causes of abdominal pain in the gastroenterology (7). Acute PVST is a rare disease that can also present with varying degrees of abdominal pain, which depends on the location and grade of venous occlusion. Portal hypertension develops when PVST progresses to a chronic stage (8).

PVST is often secondary to a single or multiple risk factors according to the Virchow's triad. Systemic risk factors, such as myeloproliferative neoplasms, prothrombin gene G20210A mutation, antiphospholipid syndrome, and local risk factors, such as intra-abdominal surgery and inflammatory conditions, are well recognized in non-cirrhotic PVST, but were not found in our case (2, 3). Notably, a history of COVID-19 was discovered in our case. Incidence of splanchnic vein thrombosis in patients with COVID-19 is about 0.6% (9). It has been studied that severe acute respiratory syndrome coronavirus 2 (SARS-CoV-2), the culprit for COVID-19, could be associated with splanchnic vein thrombosis (10). SARS-CoV-2 can bind to angiotensin-converting enzyme 2 receptors on epithelial cells, thereby leading to vascular endothelial dysfunction. Additionally, immune response secondary to infection can release a large number of inflammatory factors, such as interleukin-2, interleukin-6, and tumor necrosis factor, which can cause hypercoagulability and hypo-fibrinolysis state (11, 12).

PAI-1 plays an important role in fibrinolysis system. Elevated PAI-1 generally promotes the development of venous thrombosis by inhibiting t-PA and urinary type plasminogen activator (u-PA) (13). PAI-1 gene is located on human chromosome 7, and the 4G allele binds to enhancer region, which will increase the transcription of PAI-1 gene and the PAI-1 level (14). Genetic polymorphism is theoretically associated with splanchnic vein thrombosis, but it is still disputed. PAI-1 4G/5G may increase the risk of venous thrombosis compared with PAI-1 5G/5G (15). However, gene polymorphism may not increase the risk of splanchnic vein thrombosis, despite 4G allele is associated with inherited and acquired thrombophilia (16).

Imaging tests are needed for a definite diagnosis of PVST in patients who present with abdominal pain and have potential risk factors for PVST (17). Ultrasound is the first-line imaging method, but the accuracy of diagnosing thrombosis within mesenteric veins may be affected by ascites, obesity, and bowel gas (1, 17). Contrast-enhanced cross-sectional imaging tests can further diagnose PVST who have failed by ultrasound, and can identify the presence of cirrhosis and malignant tumors (18).

Severe PVST may lead to intestinal ischemia and necrosis, and even death (19). In order to avoid PVST related complications with their high mortality, early anticoagulation therapy has been recommended and should be maintained for at least 6 months (4, 19). Thus, our patient received anticoagulation immediately when PVST was diagnosed. Thrombolysis is considered to further improve revascularization, and its efficacy and safety have been discussed in previous studies (20–22). Local thrombolysis is preferred, and streptokinase, urokinase, and alteplase are often selected (23). As previously reported by our group, urokinase was given in patients with acute symptomatic PVST who did not respond to anticoagulation alone (24, 25). It is worth to note that we were the first to report the use of thrombolytic therapy for acute PVST by tenecteplase. We chose the dosage of tenecteplase according to the design of a Chinese study about its pharmacokinetics and tolerability, which suggested that tenecteplase at a dosage of 15–20 mg should be effective and safe (26). Compared with alteplase, tenecteplase has longer half-life and higher fibrin specificity, and is more resistant to PAI-1, which can enhance clots degradation (27). Unfortunately, our patient did not achieve venous revascularization by tenecteplase during hospitalization, which might be associated with increased fibrinolytic resistance secondary to COVID-19 (28). It could be explained by the potential mechanism that COVID-19 causes inflammatory factor storm, increasing the PAI-1 level, which is a hypo-fibrinolysis state, and then solidifying the clot structure (29). Notably, it is seemingly counterintuitive that our patient had a high level of D-dimer, which might be due to adequate tissue-level hyperfibrinolysis but insufficient systemic hyperfibrinolysis (30). Additionally, it has been shown that tenecteplase could overcome fibrinolytic resistance (29), but our patient was infected with SARS-CoV-2 during the period of acute thrombosis, which might offset the benefit of tenecteplase on fibrinolytic resistance.

Our patient had hematuria during thrombolytic period and adverse reaction disappeared by discontinuing tenecteplase. Patients treated with tenecteplase had a lower rate of non-cerebral bleeding than those treated with alteplase, and genitourinary bleeding rate in 24 h and 30 days was 0.26% and 0.38%, respectively (31, 32). However, these data were derived from AMI patients. Therefore, our case provided new data regarding use of tenecteplase for acute extensive PVST. In addition, continuously intravenous infusion of tenecteplase as a mode of administration in this patient might be a potential cause of bleeding event.

In conclusion, the probability of acute PVST should not be neglected in previously healthy patients presenting with acute abdominal pain. Considering serious complications of acute PVST, early antithrombotic treatment is needed. To the best of our knowledge, this case should be the first to use tenecteplase for the treatment of acute PVST. However, we have to acknowledge that the risk of bleeding during the continuous intravenous infusion of tenecteplase should be cautiously concerned even in the case of no definite risk factors of bleeding. In future, large-scale studies should be necessary to evaluate the optimal dosage and duration of tenecteplase in such patients and clarify how to monitor and manage its adverse effects in clinical practice.

## Data Availability

The original contributions presented in the study are included in the article/Supplementary Material, further inquiries can be directed to the corresponding author.
